# The Generation of Dynein Networks by Multi-Layered Regulation and Their Implication in Cell Division

**DOI:** 10.3389/fcell.2020.00022

**Published:** 2020-01-31

**Authors:** Takayuki Torisawa, Akatsuki Kimura

**Affiliations:** ^1^Cell Architecture Laboratory, National Institute of Genetics, Mishima, Japan; ^2^Department of Genetics, The Graduate University for Advanced Studies, SOKENDAI, Mishima, Japan

**Keywords:** cytoplasmic dynein-1, microtubule, motor activity, *C. elegans*, centrosome positioning

## Abstract

Cytoplasmic dynein-1 (hereafter referred to as dynein) is a major microtubule-based motor critical for cell division. Dynein is essential for the formation and positioning of the mitotic spindle as well as the transport of various cargos in the cell. A striking feature of dynein is that, despite having a wide variety of functions, the catalytic subunit is coded in a single gene. To perform various cellular activities, there seem to be different types of dynein that share a common catalytic subunit. In this review, we will refer to the different kinds of dynein as “dyneins.” This review attempts to classify the mechanisms underlying the emergence of multiple dyneins into four layers. Inside a cell, multiple dyneins generated through the multi-layered regulations interact with each other to form a network of dyneins. These dynein networks may be responsible for the accurate regulation of cellular activities, including cell division. How these networks function inside a cell, with a focus on the early embryogenesis of *Caenorhabditis elegans* embryos, is discussed, as well as future directions for the integration of our understanding of molecular layering to understand the totality of dynein’s function in living cells.

## Introduction

Cell division is a dynamic process in which sister chromosomes are separated into two daughter cells, and the cytoplasm is partitioned into two. In eukaryotic cells, microtubules are a major component of the cytoskeleton responsible for chromosome separation, whereas the actin cytoskeleton is mainly responsible for cytokinesis. Microtubules also play critical roles in various spatiotemporal dynamics inside the cell, together with a variety of associated proteins, including polymerization regulating factors, crosslinkers, and molecular motors (Mimori-Kiyosue, 2011; de Forges et al., 2012; Bodakuntla et al., 2019). Dynein and kinesin are two classes of molecular motor that move along microtubules (Vale, 2003). In many eukaryotic cells, cytoplasmic dynein is responsible for most of the minus-end directed motion inside the cell (Pfister et al., 2006; Wickstead and Gull, 2007; Kardon and Vale, 2009; Roberts et al., 2013), whereas kinesin is mainly responsible for plus-end directed motion. Cytoplasmic dynein-1 transports several types of cargos, such as vesicles, membranous organelles, mRNA, and viruses (Dodding and Way, 2011; Reck-Peterson et al., 2018), whereas cytoplasmic dynein-2 is responsible for retrograde transport in the cilia and flagella (Hou and Witman, 2015; Roberts, 2018). A striking feature of cytoplasmic dynein-1 is that, despite the wide variety of its function, the heavy chain polypeptide (catalytic subunit) of cytoplasmic dynein-1 is coded in a single gene (Pfister et al., 2006). In contrast, kinesin has many isoforms that perform different functions (Hirokawa et al., 2009, 2010). Kinesin isoforms are differentially expressed according to the cell cycle, whereas a single heavy chain of dynein is responsible for a variety of cellular processes throughout the cell cycle (Kobayashi and Murayama, 2009). An increasing understanding of cytoplasmic dynein-1 suggests the existence of various kinds of dynein with different molecular compositions and functions share a common catalytic subunit (heavy chain) to achieve various cellular activities (Cianfrocco et al., 2015; Reck-Peterson et al., 2018; Roberts, 2018; Olenick and Holzbaur, 2019). In this review, we refer to the various kinds of cytoplasmic dynein-1 as “dyneins.” While some dyneins transport cargos, others act as an anchor of microtubules to achieve the proper positioning of Golgi, centrosomes, and nucleus (Corthesy-Theulaz et al., 1992; Lele et al., 2018). In addition, some dyneins have a role in organizing higher-order microtubule structures, such as the spindle (Heald et al., 1996; Merdes et al., 1996, 2000; Mitchison et al., 2013).

In this review, we attempted to classify the mechanisms underlying the emergence of multiple dyneins into several layers. The layers of regulation are summarized as follows: layer 1, intra-molecular regulations within the catalytic subunit; layer 2, the control of subunit composition and integrity as a protein complex; layer 3, the regulation by accessory proteins modulating the catalytic activity, processivity, and/or cellular localizations. In addition, in layer 4, we would like to discuss the regulations mediated by forces applied to dyneins.

Inside a cell, multiple dyneins are generated through the multi-layered regulations, interacting with each other to form a network of dyneins. Interactions between dyneins are mediated by microtubules, cargo associated with different dyneins, and indirect hydrodynamic interactions. Dynein networks may be responsible for the precise regulation of cellular activities, including cell division. We will now discuss how dynein networks function inside a cell, with a focus on early embryogenesis in *Caenorhabditis elegans*.

## Layers

### Layer 1: Molecular Composition and Intramolecular Regulation of Dynein

**FIGURE 1:**
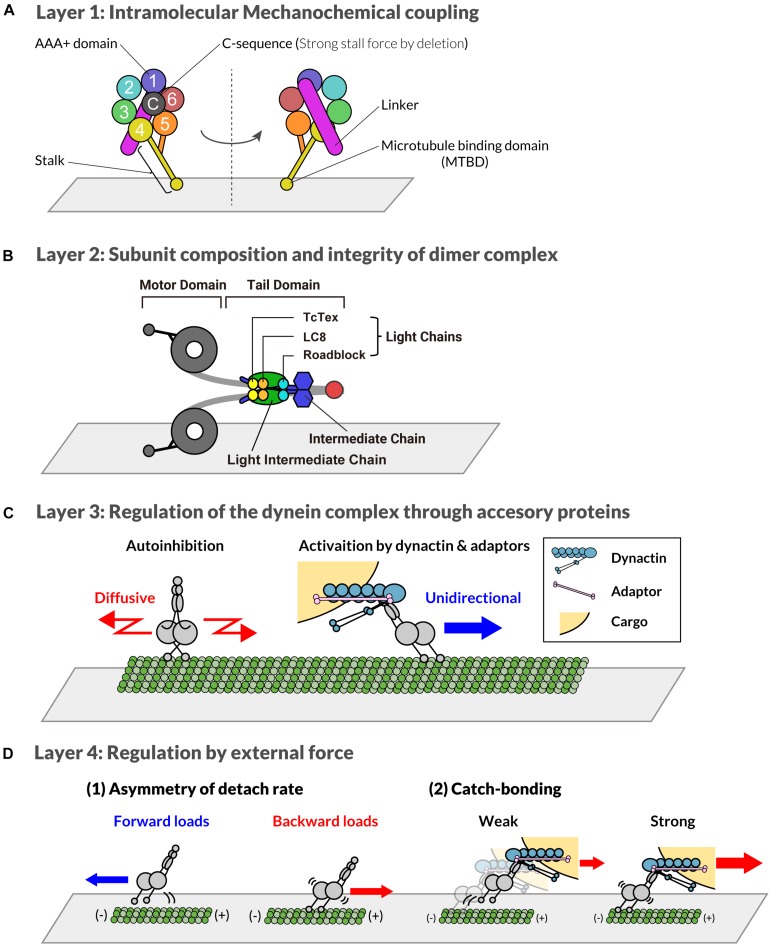
Multi-layered regulatory mechanism of cytoplasmic dynein-1. **(A)** Layer 1: distinct regions of the C-terminal motor domain of dynein heavy chain subunit are shown with different colors. The number shows distinct AAA motifs. “C” indicates the C-sequence. The stalk and microtubule binding domain extending from AAA4 are shown in yellow. The linker connected to AAA1 is depicted as a purple bar. **(B)** Layer 2: distinct subunits of dynein complex are shown with different colors. Red represents the dimerization domain of the heavy chain. **(C)** Layer 3: Through the interaction with adaptor proteins, the localization and activity of dynein is regulated. A single complex of dynein that forms a phi-particle is auto-inhibited and diffuses along the microtubule (left). In contrast, dynein associated with dynactin and adaptor protein moves unidirectionally (right). **(D)** Layer 4: Dynein activity is also regulated by forces. When dynein is pulled toward the plus-end of microtubules, it binds more strongly to microtubules compared to dynein without force or pulled toward the minus-end **(1)**. Additionally, the dynein dissociation rate decreases in the presence of high loads **(2)**.

Dynein is a multi-subunit protein complex composed of heavy, intermediate, light intermediate, and light chains (Pfister et al., 2006). Among the subunits, the heavy chain is responsible for the catalytic activity needed for force production. The heavy chain of dynein consists of an N-terminal tail region and a C-terminal motor region. The heavy chain belongs to the AAA+ superfamily of ATPases, and the six AAA domains form a ring-structured motor domain (Figure 1A; Vale, 2003). The six AAA domains have different functions. Four of the six AAA domains (AAA1-4) can bind/hydrolyze ATP (Gibbons et al., 1991; Kon et al., 2004, 2012; Carter et al., 2011; Schmidt et al., 2012), whereas AAA5 and AAA6 are only structural (Kon et al., 2012; Schmidt et al., 2012). ATP hydrolysis at AAA1 plays a major role in the motility of dynein (Gibbons et al., 1987; Silvanovich et al., 2003; Kon et al., 2004, 2005; Cho et al., 2008). ATP hydrolysis at AAA1 and the associated conformational changes of the AAA ring, induces helix sliding in the coiled-coil stalk, which extends from AAA4 (Kon et al., 2012; Schmidt et al., 2012). The helix sliding results in changes in the affinity of the microtubule-binding domain located at the tip of stalk (Kon et al., 2009; Niekamp et al., 2019). Mutagenic studies have revealed that AAA3 is involved in the ATP-mediated release of the microtubule-binding domain from microtubules, which is essential for cyclic stepping of dynein (Silvanovich et al., 2003; DeWitt et al., 2015; Nicholas et al., 2015a).

Another important domain in the C-terminal region is the “C-sequence,” which is conserved in many eukaryotic cells (Numata et al., 2011; Nicholas et al., 2015b). The C-sequence exists downstream of AAA6 and interacts with the AAA1, AAA5, and AAA6 domains (Numata et al., 2011; Kon et al., 2012; Nicholas et al., 2015b). Dynein of *Saccharomyces cerevisiae*, which generates a higher stall force than mammalian dyneins (5∼7 pN for yeast and 1–2 pN for mammalian), lacks this C-sequence. A study using an artificial dimer of mouse dynein, which contains only motor domains and linkers, reported that the deletion of the C-sequence induced the generation of a strong stall force comparable to that of yeast dynein (Nicholas et al., 2015b). However, the deletion of C-sequence had little effect on the velocity, and instead reduced the levels of microtubule-stimulated ATPase activity. These results suggest that force generation and velocity may be the controlled separately, however, a further examination of the effect of C-sequence deletion on the full-length dynein complex is needed.

The N-terminal region of the heavy chain provides a platform for interaction with other subunits of the dynein complex, which is discussed in the next section (Layer 2). Between the C-terminal motor region and the N-terminal tail region, a linker region exists. The conformational change of the linker coupled with the ATP hydrolysis cycle is required for the stepping of dynein (Kon et al., 2005; Mogami et al., 2007; Roberts et al., 2009, 2012).

### Layer 2: Subunit Composition and Integrity of Dynein Complex

The other subunits of the dynein complex, namely the intermediate, light intermediate, and three types of the light chain subunits bind directly or indirectly to the heavy chain subunit through the N-terminal tail region of the heavy chain (Figure 1B; Pfister et al., 2006; Chowdhury et al., 2015; Zhang et al., 2017). The structural core of the complex consists of a dimer of two heavy chains. These chains form a dimer through a dimerization domain in the N-terminal region (Urnavicius et al., 2015; Zhang et al., 2017). The dimerized heavy chains bind to the light intermediate chains and intermediate chains. The light chains are incorporated to the dynein complex by binding to the N-terminal region of the intermediate chains (Lo et al., 2001; Mok et al., 2001; Makokha et al., 2002; Susalka et al., 2002; Hall et al., 2010). All of the five non-motor subunits are each encoded by two genes in the vertebrate, whose cell-type specific expression pattern generates the specific composition of the dynein complex (Pfister et al., 2006; Pfister and Lo, 2011; Pfister, 2015). The composition of the dynein complex and its post-translational modifications are believed to determine the specificity of the cargos subsequently transported by the dynein complex (Tai et al., 2001; Pfister and Lo, 2011; Pfister, 2015). This regulation is carried out by specific interactors of the dynein complex, which will be discussed in detail in the section on layer 3. The dynactin complex, an essential regulator of dynein, binds to dynein through the N-terminal region of the intermediate chains (Karki and Holzbaur, 1995; King et al., 2003; Siglin et al., 2013).

Another important role of the non-motor subunits is the induction of the formation of a stable dimer complex of dynein. An *in vitro* reconstitution study showed that the heavy chain alone forms aggregation but not stable dimers (Trokter et al., 2012), although the heavy chain has an intrinsic dimerization domain in its N-terminus (Urnavicius et al., 2015; Zhang et al., 2017). The light intermediate chain induces the formation of a partially stable dimer complex, wherein the addition of the intermediate chain induces a fully stable complex with the dimer of the heavy chain. Although the light chains are dispensable for the stability of the dimerization of the heavy chains, their binding to intermediate chains is believed to contribute to the self-association of the intermediate chains (Hall et al., 2009; Nyarko and Barbar, 2011; Barbar and Nyarko, 2015).

After the formation of the dynein complex, the heavy chain no longer forms aggregates. The dynein complex alone, however, is not a processive motor, moving along the microtubules diffusively (Trokter et al., 2012; McKenney et al., 2014; Schlager et al., 2014a; Torisawa et al., 2014). The mammalian dynein complex on its own is known to exist in an autoinhibited conformation due to its intra-dimer interaction. This has been demonstrated by single molecule observations of the recombinant mammalian dynein complex, where its movement was characterized as being bidirectional and diffusive (Trokter et al., 2012; McKenney et al., 2014; Schlager et al., 2014a; Torisawa et al., 2014). This is in contrast to the case of yeast dynein, which exhibits robust processive movements along the microtubules (Reck-Peterson et al., 2006). Enzymatic and structural studies using human dynein have revealed that the two motor domains of a single dynein dimer form a characteristic conformation called the “phi-particle” (Torisawa et al., 2014; Zhang et al., 2017). In the phi-particle, two motor domains are held together with low affinity for microtubules. Because of this conformational restriction, phi-shaped dynein has a low affinity for dynactin, a protein that activates processive motility of dynein, and is thus autoinhibited. A mutant dynein with a defect in phi-particle formation was found to accumulate at the centrosome and the spindle pole, suggesting that phi-particle-based autoinhibition plays a role in the intracellular distribution of dynein (Zhang et al., 2017). Interestingly, recent studies have shown that cytoplasmic dynein-2, which is responsible for intraflagellar transport (IFT), forms a phi-particle-like conformation (Toropova et al., 2017; Jordan et al., 2018), suggesting that the formation of phi-particles is a conserved regulatory mechanism of dimeric dyneins (Roberts, 2018). In order to act as a processive motor, the dynein complex needs to interact with other proteins, such as the dynactin complex, as will be explained in the following section on layer 3.

### Layer 3: Regulation of the Dynein Complex Through Accessory Proteins

The third layer of regulation is achieved through the interaction of the dynein complex with various accessory proteins (Figure 1C). The non-motor subunits of the dynein complex are responsible for the binding of various accessory proteins. The N-terminal region of the intermediate chains binds to the dynactin complex (Karki and Holzbaur, 1995; Vaughan and Vallee, 1995; King et al., 2003; Siglin et al., 2013), a major/ubiquitous binding partner of dynein required for processive movements *in vitro* and *in vivo*. The interaction between dynactin and the intermediate chains is reduced by the phosphorylation of the intermediate chain in an isoform-dependent manner (Vaughan et al., 2001; Jie et al., 2017). The C-terminal region of the light intermediate chains binds to various “adaptor” proteins, recruiting dynein to specific cellular locations, including Spindly, bicaudal D homolog 2 (BICD2), Hook homologs, Rab interacting lysosomal protein (RILP), Rab11 family interacting protein 3 (RAB11FIP3), ninein, and TRAK1 (Schroeder and Vale, 2016; Redwine et al., 2017; Lee et al., 2018; Celestino et al., 2019). The adaptor proteins control the subcellular localization of dynein. NuMA is an important adaptor that recruits dynein to the cortex membrane (Woodard et al., 2010; Kiyomitsu and Cheeseman, 2012, 2013) and spindle poles (Merdes et al., 1996, 2000; Elting et al., 2014; Hueschen et al., 2017, 2019). The cortical-localized NuMA forms the cortical force generating machinery (Kiyomitsu and Cheeseman, 2012; Kiyomitsu and Cheeseman, 2013; Okumura et al., 2018; Kiyomitsu, 2019). The recruitment of dynein to the plus end of the microtubules depends on dynactin and EBs, and contributes to the initiation of transport (Moughamian and Holzbaur, 2012; Barbosa et al., 2017; Jha et al., 2017). HOOK proteins recruit dynein to the early endosomes and nuclear envelope (Bielska et al., 2014; Zhang et al., 2014; Guo et al., 2016; Dwivedi et al., 2019). The *C. elegans* hook protein, ZYG-12, mediates the essential attachment between the centrosome and nucleus (Malone et al., 2003; Minn et al., 2009), and thus plays a critical role in transporting the centrosome/nucleus complex. Many other adaptors and their role in the spatial control of dynein are well summarized in the literature (Liu, 2017; Olenick and Holzbaur, 2019).

Adaptor proteins not only specify the subcellular localization of dynein, but also play an important role in regulating its activity. The dynein complex alone, without adaptor proteins, forms a phi-particle and is autoinhibited (Torisawa et al., 2014; Zhang et al., 2017). Although a single molecule of purified dynein is trapped in an autoinhibited state and diffuses along a microtubule (Trokter et al., 2012; McKenney et al., 2014; Schlager et al., 2014a; Torisawa et al., 2014), dynein is clearly responsible for directed transport within cells. This apparent contradiction is resolved via the aid of regulatory proteins. Cargo-adaptor proteins, such as BICD2 and HOOK, associate dynein with dynactin, which activates the processive motility of dynein. The dynein-dynactin-adaptor complex allows for the robust processive movements (McKenney et al., 2014; Schlager et al., 2014a; Olenick et al., 2016; Olenick and Holzbaur, 2019). While dynein-dynactin-BICD2 complex moves along MTs in a processive way, Kobayashi et al. (2017) reported that CC1, one of the coiled-coil regions in the p150 subunit of dynactin, induces the dissociation of dynein and dynactin from microtubules and negatively regulates the motility of dynein. In addition, several recent studies have shown that dynein-activating cargo-adaptor proteins are normally in an autoinhibited state, and only become activated after cargo binds (McClintock et al., 2018; Sladewski et al., 2018; Noell et al., 2019).

Although complex formation of dynein with dynactin and adaptor proteins induces the robust processive movement, both *in vitro* and *in vivo*, required for the directed intracellular transport, the difference between the velocity of movement *in vitro* and *in vivo* remains to be elucidated. Whereas the dynein-dynacin-BICD2 complex moves with the velocity of several hundred nm/s to 1 μm/s (McKenney et al., 2014; Schlager et al., 2014a; Belyy et al., 2016; Olenick et al., 2016; Redwine et al., 2017; McClintock et al., 2018; Sladewski et al., 2018), the dynein-driven intracellular transport exhibits a velocity of several μm/s (Splinter et al., 2012; Schlager et al., 2014b). A rapid unidirectional transport with the velocity of several μm/s was not observed even in the team of up to eight dyneins, although the motors were clustered on the DNA nanotube (Torisawa et al., 2014). Previous studies have indicated the weak-additive nature of the velocity of dynein, suggesting the number of motors alone cannot explain the velocity of dynein-based transport. In addition, the difference in velocity between vesicles *in vivo* and purified vesicles *in vitro* suggests that the cargo-related geometrical factor is not sufficient to explain the difference in velocity (Hendricks et al., 2010). Considering that physical properties, such as ionic strength, viscoelasticity, non-equilibrium fluctuations, and hydrodynamic flow differ *in vitro* and *in vivo* (Caspi et al., 2000; Brangwynne et al., 2008; Luby-Phelps, 2013; Guo et al., 2014; Goldstein and van de Meent, 2015), further studies focusing on the effects of these factors are required to understand how rapid intracellular transport is achieved.

### Layer 4: Regulation by External Forces

Interestingly, dyneins with identical subunit compositions and identical associated proteins do not always have the same motor activity, indicating another layer of regulation. The activity of dynein is also regulated by applied forces (Figure 1D). Several biophysical studies have shown that dynein has a characteristic force response property: it binds to a microtubule more strongly in the presence of backward loads than forward loads (Cleary et al., 2014; Nicholas et al., 2015a; Rao et al., 2019). Recently, Rao et al. (2019) revealed that the asymmetric force response to directional loads is mediated by the sliding of the coiled-coils of the stalk, and that coordinated conformational changes of linker regions control this process. In addition to the asymmetry, dynein has a catch-bonding property: the unbinding rate of dynein decreases in the presence of strong loads in some force ranges (Kunwar et al., 2011; Leidel et al., 2012). Theoretical studies have argued that the force response of dynein supposed to be important for coordinated transport (Soppina et al., 2009; Bhat and Gopalakrishnan, 2012; Puri et al., 2019). Further biophysical and theoretical studies that focus on how the multiple motors share loads will be required to determine how force response regulates dynein in a team of motors.

Inside the cell, such external forces are applied to dynein through other motor proteins, applying forces to the microtubule associated with the dynein, or associated with the common cargos (see the following section). Forces may also be generated as viscous drag against dynein itself or associated cargos or microtubules.

## Network of Dyneins

In a cellular context, the dynein complex does not work alone. Multiple dyneins are connected directly or indirectly in the cell through molecular or physical interactions to form a functional network of dyneins. For example, intracellular vesicles usually contain multiple dyneins (Hendricks et al., 2010; Encalada et al., 2011; Rai et al., 2013, 2016; De Rossi et al., 2017; Chowdary et al., 2018; Cella Zanacchi et al., 2019), and the clustering enables a team of dyneins to produce collective large stall forces (Mallik et al., 2013; Rai et al., 2013). A recent super-resolution study also reported that dynein forms nanoclusters composed of up to seven dimers on microtubules (Cella Zanacchi et al., 2019). Clustering of dyneins anchored at the cortex is activated by the adaptor NuMA/Num1. Disruption of dynein clustering results in impaired spindle positioning (Tang et al., 2012; Kraft and Lackner, 2017, 2019; Okumura et al., 2018; Schmit et al., 2018), suggesting that controlling the number of motors plays a key role in dynein force generation at the cell cortex. The clustering of dynein may also play an important role in intracellular transport. Recent cryo-EM studies reveal that some activating adaptors, including BICDR1 and HOOK3, recruit two dyneins to the activated complex (Grotjahn et al., 2018; Urnavicius et al., 2018), resulting in larger stall forces and faster velocities. In addition to the clustering of dynein itself, the intracellular cargos also have the opposite-directed kinesin motors (Hirokawa et al., 1990; Martin et al., 1999; Gross et al., 2002; Ling et al., 2004; Kural et al., 2005; Pilling et al., 2006; Barkus et al., 2008; Ally et al., 2009; Soppina et al., 2009; Hendricks et al., 2010, 2012; Encalada et al., 2011; Hancock, 2014; Kendrick et al., 2019). Interestingly, several researches reported that reducing the opposite-directed motor impaired dynein-dependent transport (Martin et al., 1999; Gross et al., 2002; Ling et al., 2004; Pilling et al., 2006; Barkus et al., 2008; Ally et al., 2009; Encalada et al., 2011; Hendricks et al., 2012). These observations suggest the cooperative activation of team of motors, although the detailed mechanism of cooperative activation remains unclear. The force response property of dynein discussed at layer 4 may play a key role in cooperative transport (Soppina et al., 2009; Bhat and Gopalakrishnan, 2012; Puri et al., 2019).

The clustering of dynein also induces the formation of higher-order structures, such as mitotic/meiotic spindles. Whereas dynein contributes to the focusing of microtubules at spindle poles (Verde et al., 1991; Heald et al., 1996, 1997; Merdes et al., 2000; Goshima et al., 2005; Morales-Mulia and Scholey, 2005; Raaijmakers et al., 2013; Dwivedi et al., 2019), an *in vitro* reconstituted study demonstrated that dynein-dynactin-BICD2 complexes can organize the aster MT array through clustering at the minus end of microtubules (Tan et al., 2018). The accumulation of dynein-dynactin-BICD2 complex is regulated by the tyrosination of the C-terminus of α-tubulin, which affects the affinity of dynactin complex to microtubules (McKenney et al., 2016; Barbosa et al., 2017).

Many previous studies have shown that the distribution of dynein in the cytoplasm is highly homogeneous in many eukaryotic cells (Schmidt et al., 2005, 2017; Nguyen-Ngoc et al., 2007; Gassmann et al., 2008; Kobayashi and Murayama, 2009; Kimura and Kimura, 2011; Fielmich et al., 2018; Rodriguez-Garcia et al., 2018). As discussed in the section on layer 3, in the absence of adaptor proteins, the dynein complex is in the auto-inhibited state (Torisawa et al., 2014; Zhang et al., 2017). Active dynein accumulates at the minus-end of microtubules, while auto-inhibited dynein is distributed uniformly in the cytoplasm, indicating that dynein activity is regulated, not by dynein gene expression, but by the distribution of dynein regulatory proteins. In fact, a recent study suggested that the force imbalance in the first cell division in *C. elegans* embryos is caused by the asymmetric distribution of activating adaptors on the cell cortex, not by the distribution of dynein (Rodriguez-Garcia et al., 2018).

## The First Cell Division of *Caenorhabditis elegans*, as a Model for the Functional Networks of Dyneins

So far, we have discussed that the multi-layered regulation of cytoplasmic dynein can generate multiple types of dynein (i.e., dyneins) with distinct biochemical and biophysical activities. These dyneins may contribute to provide diversity to the cellular toolbox of minus-end-directed microtubule motors. Furthermore, multiple dyneins may act cooperatively to function as a “network of dyneins” to accomplish complicated cellular activities.

Examples of multiple dyneins acting as a network include the migration of neurons and the formation of the mitotic spindle. In migrating neurons, a population of cytoplasmic dyneins in the migratory process pulls the centrosome in the direction of migration. Meanwhile, another dynein population on the nuclear surface pulls the nucleus toward the centrosomes. The combined action of these two dynein populations moves the nucleus in migrating neurons (Tsai et al., 2007; Vallee et al., 2009). In dividing cells, the formation of the mitotic spindle requires multiple dyneins functioning at various locations (Prosser and Pelletier, 2017). At the poles of the spindle, dynein is involved in pole focusing. At the kinetochore, dynein aligns the chromosomes. Dynein also slides along the microtubules to generate forces required for the integrity of the spindle structure. Dynein at these different locations appears to possess different subunit compositions and/or to associate with different proteins (Raaijmakers et al., 2013). These examples demonstrate that multi-layered regulation of dyneins is critical to certain *in vivo* processes.

In describing how multiple dyneins act as a network, we use the first cell division of *C. elegans* as model system. In this system, the dynamic reorganization of the cell occurs in less than 1 h after fertilization (Pintard and Bowerman, 2019). Cytoplasmic dynein functions in various processes, such as the formation of meiotic and mitotic spindles, and the positioning of the nucleus and spindle (Table 1). Because the cytoplasmic dynein is responsible for various functions, we assume cells generate multiple dyneins through multi-layered regulation in this system. In fact, the localization, cargos, enzymatic activities, and temporal changes of dynein are different for each process, suggesting the coexistence of multiple dyneins in the cell. At the same time, the dyneins share a common space (i.e., cytoplasm) and time, suggesting that they may influence each other and function as a network. Therefore, we believe that the first cell division of *C. elegans* is a good model to examine the function a network of dyneins *in vivo*.

**TABLE 1:** Processes involving dynein in *C. elegans* one-cell embryos.

Processes (*1)	Localization of dynein in action	Other subunit involved	Accessory proteins involved	References
Oocyte meiotic spindle formation				Gonczy et al., 1999
Attachment of the centrosomes to the sperm pronucleus	Nuclear surface		ZYG-12, SUN-1	Gonczy et al., 1999; Malone et al., 2003
Centrosome separation	Nuclear surface, Cell cortex		ZYG-12, SUN-1, GPA-16, GOA-1	Gonczy et al., 1999; Malone et al., 2003; De Simone et al., 2018
Oocyte pronuclear migration	Nuclear surface	DNC-1, DNC-2	ZYG-12, SUN-1	Skop and White, 1998; Gonczy et al., 1999
Sperm pronuclear migration and centering	Cytoplasm, Cell cortex (*2)	DNC-1, DNC-2, DYRB-1 LIS-1/NudE	ZYG-12, SUN-1, RILP-1	Skop and White, 1998; Gonczy et al., 1999; Cockell et al., 2004; Kimura and Onami, 2005; Goulding et al., 2007; Kimura and Kimura, 2011
Nuclear rotation	Cell cortex, Cytoplasm	DNC-1, DNC-2		Skop and White, 1998; Gonczy et al., 1999; Kimura and Onami, 2007
Mitotic spindle formation		LIS-1/NudE	SPDL-1, Rod/Zwilch/Zw10	Gassmann et al., 2008; Simoes et al., 2018
Spindle displacement	Cell cortex	DYRB-1	PAR-2, PAR-3, GPA-16, GOA-1, GPR-1, GPR-2, LIN-5	Grill et al., 2001; Colombo et al., 2003; Couwenbergs et al., 2007; Nguyen-Ngoc et al., 2007; Fielmich et al., 2018
Spindle rocking	Cell cortex		GPA-16, GOA-1, GPR-1, GPR-2	
Spindle elongation	Cell cortex		GPA-16, GOA-1, GPR-1, GPR-2	

*1, In temporal order; *2, Antagonistic role, see text.

**FIGURE 2:**
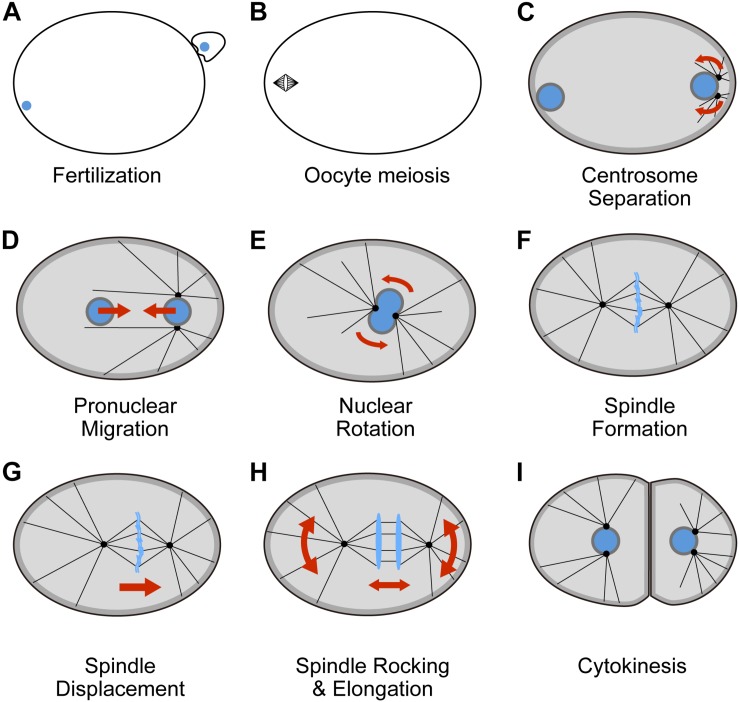
Intracellular localization and function of dynein during the first cell division of *C. elegans* one-cell embryo (**A–I**, in temporal order, see also text). Intracellular localization of the heavy chain subunit of dynein (DHC-1) is shown in dark and light gray (Gonczy et al., 1999). Nuclei and chromosomes are shown in blue. The movement of the centrosomes driven by dynein are denoted by red arrows. See Table 1 for the references describing the involvement of dynein in each process.

In this review, we focus on centrosome positioning during the first cell division of *C. elegans* (Figure 2). After fertilization (Figure 2A), the two centrosomes are formed in the vicinity of sperm pronucleus, as the centrioles are provided to the embryo by the sperm. The two centrosomes are separated into the two poles of the sperm pronucleus (Figure 2C). After separation, the centrosomes move toward the cell center together with the sperm pronucleus, and later also with the oocyte pronucleus after pronuclear meeting (Figure 2D). The line connecting the two centrosomes is first perpendicular to the long axis of the embryo, but later rotates to become parallel to the long axis by the time of the centrosomes reach the cell center (Figure 2E). After nuclear envelope breakdown (NEBD), the mitotic spindle is formed and the centrosomes become the two poles of the spindle (Figure 2F). The centrosomes, together with the mitotic spindle, will be displaced toward the posterior pole to prepare for an asymmetric cell division (Figure 2G). The spindle also oscillates perpendicular to the long axis, as the two centrosomes separates further and thus the spindle elongates (Figure 2H). Three distinct types of dyneins, at least, seem to exist in the cell and they cooperatively move the centrosomes in the dynamic and regulated manner.

### Multiple Dyneins for Centrosome Positioning in the *C. elegans* One-Cell Embryo

The catalytic subunit of cytoplasmic dynein (DHC-1) localizes to the cytoplasm, cell cortex, and nuclear surface (Gonczy et al., 1999). The dynein in these three distinct localizations can be considered to possess distinct biochemical and biophysical properties, and can thus be considered as three different types of dyneins. The anchoring mechanisms for these three distinct types are well understood (i.e., layer 3 regulation). The localization to the nuclear surface requires SUN-1 and ZYG-12 proteins that forms a SUN/KASH pair, a well-conserved protein complex penetrating through the inner and outer nuclear membrane (Malone et al., 2003; Hiraoka and Dernburg, 2009). The localization of the cell cortex is mediated through trimeric G protein complexes (GOA-1 and GPA-16 as the alpha subunits), GPR-1 and GPR-2 proteins, and LIN-5 protein (Nguyen-Ngoc et al., 2007). The localization mechanisms at the cytoplasm are less clear. In addition to a uniform distribution over the entire cytoplasm, possibly as an inactive phi-particle (see the section on layer 2), dynein should localize at the surface of organelles. Several organelles move toward the minus end of microtubules in a dynein-dependent manner (Gonczy et al., 1999; Kimura and Kimura, 2011). RILP-1 is a protein known to connect dynein and lysosomes in mammalian cells through light intermediate chains (Celestino et al., 2019), and required to the lysosome movement in the one-cell stage embryo of *C. elegans* (Kimura and Kimura, 2011), indicating that RILP-1 is mediating organelle localization of dynein.

The subunit composition may be different (layer 2 regulation). DYRB-1 is not required for centrosome separation on the nuclear surface, but is required for the forces produced in the cytoplasm and cell cortex (Couwenbergs et al., 2007; Kimura and Kimura, 2011). Point mutants of dynein show distinct enzymatic activities of dynein (layer 1 regulation) are responsible for the three distinct “dyneins.” Temperature-sensitive mutations identified for the *dhc-1* gene cause defective spindle formation but have no effect on centrosome positioning. For example, a temperature sensitive mutation (*or195* allele) in the CC2 domain of the coiled-coil stalk results in spindle shortening and failure in cytokinesis (Schmidt et al., 2005). The suppressors of the point mutants of *dhc-1* have been identified, supporting a regulation of dynein activity through dynein subunits and adaptor proteins (O’Rourke et al., 2007).

### Network of Dyneins for Centrosome Positioning in the *C. elegans* One-Cell Embryo

In the previous section, we listed dyneins in the cytoplasm, cortex, and nuclear surface as distinct dyneins. These dyneins cooperatively function in various aspects of centrosome positioning and thus can function as a network of dyneins.

The first process is centrosome separation, in which a pair of the centrosomes separate into two poles of the sperm pronucleus. Dynein on the nuclear surface, anchored by SUN-1/ZYG-12 (Malone et al., 2003), slides the microtubules connected to the centrosomes to move the centrosomes (Gonczy et al., 1999). Dynein at the cell cortex also contributes to centrosome separation. The dynein at the cortex pulls the centrosomes via microtubules (“cortical pulling force”) and separates the centrosomes through a nucleus-independent manner (Malone et al., 2003). The cortical pulling force separates the centrosomes along the nuclear surface also in *Drosophila* embryo (Cytrynbaum et al., 2003). Dyneins on the nuclear surface and at the cortex both contribute to centrosome separation, where the knockdown of both functions results in defects in separation (De Simone et al., 2018).

The second process is nuclear centration. After centrosome separation, the centrosomes move toward the center of the cell. The sperm pronucleus and the oocyte pronucleus (after the two pronuclei meet) are anchored to the centrosomes via the dynein on the nuclear surface. With this mechanism, the pronuclei also move to the center. Nuclear centration depends on dynein as a knockdown of DHC-1 by RNAi, resulting in a complete lack of the centration (Gonczy et al., 1999; Kimura and Onami, 2005). From this and other arguments, it is difficult to explain the centration by dynein-independent forces, such as the pushing force generated by the polymerization of microtubules (Kimura and Onami, 2005). Instead, several lines of evidence suggest that dyneins in the cytoplasm contribute positively to centration (Kimura and Onami, 2005, 2007; Kimura and Kimura, 2011). Dynein at the cell cortex is also active during centration but contributes negatively by pulling the centrosomes and nuclei backward toward the nearest cortex (Kimura and Onami, 2007). It should be noted here that a study reported that centration slows down after the pronuclear meeting upon inhibiting dynein at the cortex (Goulding et al., 2007), but this is likely due to the centration speeding up in this condition, which makes the meeting to occur near the center and there is little distance to travel after the meeting. In summary, for nuclear centration, dyneins at the cytoplasm and at the cell cortex act in an opposing manner.

Similarly, when the mitotic spindle displaces toward the posterior pole, the dyneins at the cortex and at the cytoplasm act in an opposing manner. In this case, dynein at the cortex produces a major force contributing positively to the movement (Grill et al., 2001; Nguyen-Ngoc et al., 2007) whereas dynein at the cytoplasm likely acts negatively to the displacement (Kimura and Onami, 2007). The mitotic spindle also shows oscillatory movement perpendicular to the long axis of the cell. The oscillation is explained by a combination of a positive feedback regulation, in which a displacement of a spindle pole to one direction enhances a force moving the pole to the same direction, and a negative feedback regulation suppresses excess displacement in one direction (Grill et al., 2005; Pecreaux et al., 2006). Dynein at the cortex is responsible for positive feedback (Pecreaux et al., 2006), whereas dynein at the cytoplasm is responsible for negative feedback (Sugioka et al., 2018). These examples also indicate that dyneins at the cytoplasm and at the cortex act cooperatively, however, in an opposing manner. It should be noted here that the involvement of dynein at the cytoplasm in spindle displacement and spindle oscillation is still under debate, wherein some studies argue that the pushing force generated by the polymerization of microtubules is likely to play a role in the process (Garzon-Coral et al., 2016).

Another example of the cooperative action of different dyneins is nuclear rotation. During nuclear centration, the line connecting the two centrosomes is initially perpendicular to the long axis of the cell, but later rotates to become parallel to the long axis. This rotation of the nucleus contributes to the alignment of the mitotic spindle along the long axis of the cell, which is known as Hertwig’s long axis rule (Minc et al., 2011). Dynein in the cytoplasm is sufficient for this rotation (Kimura and Onami, 2005, 2007). Dynein in the cellular cortex is not essential for rotation, but does cause the rotation to be earlier and faster (Kimura and Onami, 2007). Therefore, dyneins at the cytoplasm and at the cortex both act positively to the nuclear rotation. In conclusion, during dynamic centrosome positioning of the one-cell stage *C. elegans* embryo, multiple dyneins act cooperatively as a network to accomplish temporal and spatial regulation.

## Conclusion and Perspectives

In this review, we discussed the coordination of different types of “dyneins” generated by multi-layered regulatory mechanisms, including enzymatic regulation in the motor domain, the subunit compositions, the activation by the associated proteins, and the regulation by force in cellular contexts. To further understand the coordination of multiple dyneins, studies focusing on the cellular processes, that is, where dyneins interact directly or indirectly, will provide more details. As an example of such processes, we discussed that microtubule dynamics in the early embryogenesis of *C. elegans* embryos, where many types of dyneins coordinate to drive various processes in a short period of time, mainly focusing on the role of multi-layered regulatory network of dynein. In addition to the cell biological attempts, *in vitro* reconstitution of the microtubule-related cellular processes (Laan et al., 2012; Dogterom and Surrey, 2013; Vleugel et al., 2016a, b) and their expansion will help us to characterize the coordination of dyneins.

## Author Contributions

All authors listed have made a substantial, direct and intellectual contribution to the work, and approved it for publication.

## Conflict of Interest

The authors declare that the research was conducted in the absence of any commercial or financial relationships that could be construed as a potential conflict of interest.
